# Drug administration adjustments for elderly patients with dysphagia: A case report

**DOI:** 10.1590/1980-57642018dn12-010015

**Published:** 2018

**Authors:** Patrícia de Carvalho Mastroianni, Marcela Forgerini

**Affiliations:** 1 Adjunct Professor, Department of Drugs and Medicines, School of Pharmaceutical Sciences, São Paulo State University (UNESP), Araraquara, SP, Brazil; 2 Master Student of the Pharmaceutical Sciences Program, Department of Drugs and Medicines, School of Pharmaceutical Sciences, São Paulo State University (UNESP), Araraquara, SP, Brazil

**Keywords:** medication errors, medication therapy management, pharmaceutical care, cognitive dysfunction, deglutition disorder, sertraline, erros de medicação, conduta do tratamento medicamentoso, atenção farmacêutica, disfunção cognitiva, transtornos de deglutição, sertralina

## Abstract

An elderly patient, aged 76 years, diagnosed with dysphagia, depression, hypothyroidism, Alzheimer's disease and mild cognitive deficit, was identified with sertraline and levothyroxine- drug-related problems. Medication Therapy Management (MTM) was used to adjust therapy to the patient's needs by macerating sertraline tablets and solubilizing them in 10-30 mL of orange juice. The patient was advised to take levothyroxine after fasting. Six months later, pharmaceutical follow-up identified an increase in the Mini-Mental State Exam score from 22 to 26 and a decrease in the Clinical Dementia Rating (CDR) scale score from 1.0 to 0.5 in conjunction with mood and physical improvements, as well as a significant decrease in aggressiveness and agitation. Cognitive deficit may be a result of poor drug administration procedures, leading to drug ineffectiveness. Optimizing levothyroxine and sertraline administration, based on knowledge of their physicochemical properties, improves their clinical effectiveness, including the cognition of patients with Alzheimer's disease and dysphagia.

## RESUMO

Paciente idosa, 76 anos, diagnosticada com disfagia, depressão, hipotireoidismo, doença de Alzheimer e déficit cognitivo leve, foram identificados problemas relacionados com a sertralina e levotiroxina. O Gerenciamento da Terapia Medicamentosa foi usado para ajustar a terapia às necessidades da paciente, macerando comprimidos de sertralina e solubilizando-os em 10-30 mL de suco de laranja. Foi recomendado a paciente tomar levotiroxina após o jejum. Seis meses depois, o seguimento farmacêutico identificou um aumento no score da escala Mini-Mental de 22 para 26 e Avaliação Clínica da Demência (CDR) de 1,0 para 0,5 em conjunto com melhorias físicas e de humor, bem como uma diminuição significativa na agressividade e agitação. O déficit cognitivo pode ser o resultado de procedimentos de administração inadequada de fármacos, levando à ineficácia do fármaco. A otimização da administração de levotiroxina e sertralina, com conhecimento de suas propriedades físico-químicas, melhora sua efetividade clínica, incluindo a cognição do paciente com a doença de Alzheimer e disfagia.

The Brazilian elderly population has been increasing since the 1960s, leading to a growing concern regarding age-related medical problems.^1^ It is well known that ageing-associated physiological changes can modulate pharmacokinetics and pharmacodynamics, resulting in greater demands on health team care. In addition, ageing is associated with multiple disorders,^1^ including consequences of recurrent polymedication (use of two or more medicines within a period >240 days),^2^ which contributes to complex pharmacotherapy, drug interactions and other drug-related problems (DRPs).^3^ Dysphagia (swallowing difficulty) is a growing health concern in our aging population. It is defined as a combination of conditions that are related to esophagus and neurologic disorders,^4^ which may be permanent or intermittent, but lead to pharmacotherapy dependence due to medicine deglutition problems. Dysphagia is a common clinical manifestation among the elderly and an important symptom in dementia. It has been estimated that up to 45% of patients with dementia have some degree of swallowing difficulties.^5^ The prevalence of dysphagia among patients with Alzheimer's disease is about 28 to 32%^6^ and can be as high as 50% in patients with Parkinson's disease.^7^ Furthermore, dysphagia can negatively affect patient medication use,^8^ thereby influencing decisions involving patient prescriptions,^9^ consequently impacting pharmacotherapy effectiveness and safety. Therefore, dosage adjustment strategies in patients with dysphagia and dementia can result in improved clinical effectiveness and cognition.

## CASE REPORT

A retired patient, aged 76 years, weighing 65.1 kg, 1.46 m in height, with a body mass index of 30.54 kg/m^2^, and with diagnoses of hypothyroidism (five years), major depressive disorder (eleven years), Alzheimer's disease (AD) (eight years) and moderate dysphagia - Score 4, Scale ASHA-NOMS^10^ - (five years) presented at the pharmacy clinic with complaints of apathy/fatigue, agitation, aggressiveness and hallucination, according to her caregiver.

In order to approach the case systematically, the Pharmacotherapy Workup was used.^11^ This tool is approved by the São Paulo State University's Ethics Committee, and is divided into three cyclic processes: [1] Initial Assessment for pharmacotherapy evaluation and the identification of drug-related problems based upon the patient's medication experience,^12^ clinical evaluation, lab results, medicines used, clinical history and the most relevant guidelines;^13^ [2] Pharmaceutical Care Plan for therapeutic goals incorporating active patient participation for resolving DRPs; [3] Final Assessment, which checks for achievement of therapeutic goals, DRP resolution and the identification of any new DRPs. The study was approved by the local Research Ethics Committee under CAAE: 08172112.0.0000.5426.

At first assessment, the patient's Mini-Mental State Exam (MMSE) score was 22 and Clinical Dementia Rating^14^ (CDR) score was 1, indicating a mild cognitive deficit.^15^ Estimated creatinine clearance level was 81.9 mL/min, indicating normal renal and hepatic function. The patient had no previous history of alcohol or drug abuse or evidence of drug allergy or adverse events in her medical history. The patient was prescribed the following medications: donepezil, 10mg once a day, since 2007 (manufacturer: Wyeth); flunitrazepam, 1 mg once a day, since 2011 (manufacturer: Roche); levothyroxine, 38 mcg once a day, since 2010 (manufacturer: Sanofi-Aventis); losartan, 50 mg once a day (manufacturer: EMS); sertraline, 50 mg once a day, since 2007 (manufacturer: Eurofarma); memantine, 10 mg twice a day, since 2011 (manufacturer: Eurofarma); and quetiapine, 25 mg three times a day since 2011 (manufacturer: Germed). Drug-drug interactions were investigated, with the most relevant clinical interactions being: sertraline/quetiapine (CYP3A4/CYP2D6 substrates), sertraline/flurazepam (CYP3A4 substrates) and sertraline/losartan (CYP3A4 substrates). Sertraline and memantine co-administration seems to promote both efficacy and safety.

Besides drug interactions, adverse reactions to each of these drugs were investigated. Sertraline had the most relevant adverse reactions, matching the patient's clinical presentation. Hypothyroidism, fatigue, agitation/aggressiveness can be associated with sertraline use. In order to assess hypothyroidism, three samples of free thyroxine (T4) levels were collected, as well as one sample of thyroid-stimulating hormone (TSH) level. The results for the T4 samples were: first sample (six months before the pharmaceutical visit) = 1.2 ng/dl; second sample (one month before the pharmaceutical visit) = 0.96 ng/dl; third sample (near pharmaceutical visit) -0.77 ng/dl (T4 reference range for hypothyroidism: <0.89 ng/dl). The second sample provided the TSH level, which was 3.47 mcUI/mL (TSH range: 0.4 - 4.0 mcUI/mL). The caregiver was asked about how the patient's medications were administered, reporting that she solubilized the macerated sertraline tablet in yogurt, whilst levothyroxine was taken without fasting. The caregiver also indicated that there were no dose omissions during the treatment, and therefore no non-adherence problem. Moderate adjustments were required for the patient, since all medications in use need maceration.

The clinical pharmacist informed the patient's caregiver as to the ineffectiveness of levothyroxine when given under these conditions. The patient's caregiver was told to give the levothyroxine tablet with water under fasting conditions, namely in the morning, 30 minutes before breakfast, as recommended.^16^ With regard to sertraline, the caregiver was taught to macerate and solubilize the sertraline tablet in 10-30 mL of orange or lemon juice, as recommended.^17^ Over the course of this education session, the patient and her caregiver had autonomy on the decision as to whether the medication was taken according to the pharmacist's recommendation.^9^ The patient was informed that sertraline and levothyroxine may improve her health. Apart from these sertraline and levothyroxine treatment adjustments, no other drug therapy was modified. At the time of the final assessment, six months later, the recommended pharmaceutical administration changes had been incorporated. The patient reported mood and physical improvements, as well as an absence of aggressiveness and agitation. The Mini-Mental State Exam score increased from 22 to 26 and CDR scale score decreased from 1.0 to 0.5, indicating drug effectiveness for wider aspects of cognition. Finally, it was recommended that donepezil treatment should be evaluated, according to criteria of AD guidelines.^18^

The study was approved by the local Research Ethics Committee under CAAE: 08172112.0.0000.5426.

## DISCUSSION

Hypothyroidism and depression can contribute to cognitive deficits, requiring their investigation for differential diagnosis in AD presentations.^19^ In this context, the American Psychiatric Association recommends an initial assessment for vitamin B12 deficiency and hypothyroidism in patients with probable dementia.^20^ It is therefore mandatory to assess and monitor these clinical conditions, in order to ensure optimal AD treatment. Depression can be a risk factor for dementia in elderly patients, with depression's biological underpinnings also contributing to temporary cognitive deficits.^20^^,^ 21 It is important to emphasize the importance of investigating possible correlations between clinical manifestations (for example, hypothyroidism) and drug use (for example, sertraline). Patients started on sertraline need to be closely monitored for thyroid function because TSH levels may be elevated, leading to a higher thyroid hormone concentration.^22^ Fatigue, memory impairment and weight gain are commonly associated with hypothyroidism.^22^ Furthermore, it is important to closely monitor geriatric patients in the initial months of sertraline use, in order to identify behavioral changes (Zoloft package insert). Hence, we recommend sertraline level monitoring in conjunction with TSH and T4 level monitoring in the initial months of treatment in geriatric patients undergoing polypharmacy.

Dysphagia is also common among elderly patients, compromising the ingestion and absorption of food, vitamins and medicines.^8^^,^ 23 Based upon physical and chemical properties, namely the pharmacokinetics and pharmacodynamics, of sertraline and levothyroxine, it was possible to design a strategy to increase drug efficacy and the patient's adherence.

Levothyroxine bioavailability ranges from 40 to 80% when administered orally. However, its bioavailability increases under fasting condition. Levothyroxine clinical trials show that this drug is more readily absorbed by the digestive tube in the morning, and under fasting conditions, compared to oral administration near meal-times.^24^^-^ 26 Absorption of levothyroxine is pH-dependent, with gastric pH significantly determining drug dissolution. Acid-dependent dissolution contributes to further absorption of levothyroxine in the small intestine. Sertraline is highly solubilized in acid media, such as orange and lemon juices. The concentration of the non-ionized form of sertraline is increased in acid media and its liposolubility facilitates drug absorption, increasing its bioavailability and effectiveness.^17^^,^ 27 For this reason, it is strongly recommended that sertraline drops be diluted in acid media.^17^

However, the liquid form of sertraline is not widely available under the Brazilian public health system. This drug is not included in the essential drug list in Brazil, with only the tablet form of sertraline available at this point in time. In contrast, fluoxetine is available as an essential medication for depressed patients. However, it is not the safest drug in this clinical population due to the increased risk of thrombocytopenia and QTc prolongation.^17^ Consequently, given these clinical considerations, such as when available specific pharmaceuticals are not optimal for specific patient needs, as indicated here by clinical condition and age,^28^ it is imperative to make drug adjustments that are based upon known physicochemical properties and stability, especially when oral intake is compromised by dysphagia, for instance.^17^

Therefore, clinical and cognitive improvement can be attributed to the better absorption of medication, especially levothyroxine under fasting conditions, and not to the management of dysphagia alone.

As regards drug effectiveness, data on physicochemical properties, pharmacokinetics and pharmacodynamics are essential for dose adjustments. Benzi and Mastroianni have been working on many procedures for optimizing drug prescriptions that meet patient needs, in turn enhancing drug effectiveness, safety and quality.^29^

In conclusion, the health technology of Medication Therapy Management takes into consideration the baseline disease (AD), risk factors (hyperthyroidism and depression), Evidence-based Health (EBH) practices, patient and caregiver medication experiences and the rationale for the use of medications, in order to identify drug-related problems (such as medication error and drug ineffectiveness). The use of an effective clinical assessment, such as the Mini-Mental State Exam and CDR scale, allows the identification of drug-related problems. Based on this data, in conjunction with the EBH and MTM approaches, it was possible to adequately adjust drug doses, in turn promoting full patient adherence, as well as improving the efficacy and safety of the prescribed medications.

Therefore, dosage adjustment strategies in patients with dysphagia and dementia can promote clinical effectiveness and improved cognition.
